# Digital empowerment for refined surgical privileging management: a model construction and empirical study

**DOI:** 10.3389/fpubh.2026.1701167

**Published:** 2026-03-24

**Authors:** Hu Haowei, Wang Bubu, Zhang Xinxin, Zhang Binbin, Zhu Hong

**Affiliations:** The Third Affiliated Hospital of Wenzhou Medical University, Wenzhou, Zhejiang, China

**Keywords:** empirical study, information empowerment, model construction, refinement, surgical privileging management

## Abstract

**Objective:**

This study aims to develop a refined surgical privileging management model empowered by information technology. It empirically evaluates the model’s effectiveness in addressing traditional authorization challenges, such as lack of dynamic supervision, to enhance surgical safety management.

**Methods:**

This research employed a retrospective quasi-experimental (pre-post) design. A total of 79 surgeons and 3,914 surgical cases from January 2022 to December 2023 were selected to compare the new model against the traditional system. An evaluation system comprising 12 indicators across four dimensions—surgeon qualifications, surgical practice, patient safety, and interdisciplinary collaboration—was established. An integrated informatization platform was developed with functions including data collection, dynamic evaluation, monitoring and early warning, and audit trail management. A comprehensive evaluation was conducted by comparing indicators across four quarters, including process efficiency, medical quality and safety, resource utilization, system performance, and user satisfaction.

**Results:**

With continuous advances in medical technology and rapid expansion of hospital scale, the limitations of traditional extensive privileging models have become increasingly apparent. The information-empowered model constructed in this study significantly improved management efficiency, with authorization review time stabilized between 4.61 and 4.85 working days, and an information transfer completion rate reaching 95.87–96.91%. Medical quality indicators remained consistently excellent: surgical success rates ranged from 97.82 to 98.12%, complication rates from 0.16 to 0.21%, and the unplanned reoperation rate was 1.03%. The 30-day readmission rate decreased from 1.89 to 1.21%. Resource utilization metrics remained efficient, with operating room turnover rates between 84.12 and 86.58%. System user satisfaction scores reached 4.12 to 4.26 points.

**Conclusion:**

The refined surgical privileging management model based on information empowerment effectively enhances authorization efficiency, ensures patient safety, and optimizes resource allocation. It facilitates a shift from experience-based management to data-driven refinement and intelligence, demonstrating significant value for broader implementation.

## 1 Introduction

With the continuous advancement of medical technology and the rapid expansion of hospital scale, modern hospitals are facing unprecedented challenges in medical service delivery, disciplinary development, and operational management. As one of the highest-risk and most resource-intensive components of the healthcare system, the safety and standardization of surgical procedures are directly linked to patient outcomes and institutional reputation (1). However, the current surgical authorization management model remains relatively rudimentary. Previous studies indicate that traditional processes, primarily relying on static indicators such as cumulative surgical volume and professional titles, often result in prolonged review cycles exceeding 7–10 working days and an administrative error rate of approximately 5–8% due to manual data entry (2–4). This not only undermines the scientific rigor and transparency of the authorization process but also predisposes the system to medical safety risks and imbalanced resource allocation. In recent years, alongside the promotion of “Smart Hospitals” and health informatization, technologies such as big data, artificial intelligence, and information empowerment have gradually integrated into medical management practices, offering new approaches to surgical authorization (5). Information empowerment enables comprehensive collection and multi-dimensional analysis of surgery-related data. Through real-time monitoring and visual representation, it facilitates dynamic assessment of surgeons’ competency, surgical risk levels, and patient outcomes, thereby providing a scientific basis for authorization decisions (6, 7). Concurrently, while early “Smart Hospital” frameworks have reduced administrative workloads by 20–30%, they often focus on logistical workflow indicators while overlooking critical clinical safety metrics such as unplanned reoperation rates, standardized complication scores, and interdisciplinary collaboration indices (4, 8). There remains a lack of quantitative evidence demonstrating how integrated data-driven models can simultaneously optimize both process efficiency and long-term patient outcomes. Against the backdrop of frequent medical safety incidents and increasingly stringent regulatory requirements, constructing a refined surgical authorization management model based on information empowerment is both an urgent need for hospital management transformation and a critical lever for improving the quality of medical services (9). Empirical research can not only validate the applicability and operability of this model across different types of hospitals but also quantify its comprehensive impact on surgical safety, medical efficiency, and professional development of surgeons, providing a theoretical foundation and practical experience for future dissemination. Furthermore, this study contributes to advancing surgical management from a traditional experience-based approach to a data-driven paradigm, enabling the scientification, precision, and intellectualization of medical management models, thereby offering new pathways for high-quality development of hospitals in China and enhanced patient safety.

## 2 Materials and methods

### 2.1 Study design, setting, and population

#### 2.1.1 Sampling technique and sample size

This study adopted a retrospective quasi-experimental design using purposive sampling. It focused on attending surgeons at ***** Hospital from January 2022 to December 2023 to evaluate improvements compared to the pre-implementation baseline. A total of 79 surgeons from various specialties—including general surgery, orthopedics, neurosurgery, and thoracic surgery—were enrolled, contributing 3,914 surgical cases. All data were verified as complete and auditable to ensure a robust evaluation of the model.

#### 2.1.2 Inclusion criteria

① Age between 28 and 55 years (The lower limit of 28 is set to ensure all participants have completed the standardized residency training program and obtained the title of attending physician, which is the baseline for independent surgical authorization in the study setting), holding a valid medical practitioner license, and continuous service at the hospital for ≥2 years;

② Performance of ≥80 procedures (either independent or supervised) in the past 2 years, with no fewer than 40 being Grade II or higher surgeries. These volume thresholds were defined by the institutional Medical Affairs Committee based on learning curve requirements and average specialty workload to ensure a statistically representative sample for competency assessment (8). Here, surgery grades are defined by complexity and risk: Grade I (simple procedures), Grade II (moderately complex), Grade III (high complexity and risk), and Grade IV (extremely complex/major procedures with significant risk);

③ Complete surgical logs, patient records, and postoperative follow-up data, with missing data not exceeding 5%;

④ Provision of informed consent and voluntary participation.

#### 2.1.3 Exclusion criteria

① Interns, visiting scholars, and newly hired surgeons without independent operative privileges (*n* = 15);

② Surgeons with fewer than 80 total procedures or fewer than 40 major (Grade II+) surgeries (*n* = 12);

③ Those with major medical errors, intraoperative mortality >3%, or formal disciplinary action within the previous year (*n* = 8);

④ Cases with >5% missing records or insufficient data integrity for analysis (*n* = 6).

The final cohort comprised 79 attending surgeons, corresponding to 3,914 procedures. The sample included 58 male (73.4%) and 21 female (26.6%) surgeons, with a mean age of 38.7 ± 6.2 years. Case distribution was as follows: general surgery (*n* = 1,502), orthopedics (*n* = 1,023), neurosurgery (*n* = 715), and thoracic surgery (*n* = 674). This sample is representative of surgical authorization practices in mid- to large-scale general hospitals and provides a robust foundation for constructing and validating an informatics-driven refined surgical privilege management model.

### 2.2 Development of system and data collection

#### 2.2.1 Development of a multidimensional privileging criteria system

A refined privileging framework was developed following a two-round Delphi expert consultation (*n* = 15 experts) to ensure content validity. The weights for each indicator (Table 1) were then determined using the Analytic Hierarchy Process (AHP), ensuring logical consistency and mathematical rigor in the prioritization of safety over administrative metrics. In this model, “Medical License” and “Professional Title” are integrated into a single indicator because they collectively represent the non-negotiable legal and professional baseline for independent surgical practice, serving as the primary entry threshold for the privileging assessment.

**Table 1:** Multidimensional refined privileging criteria system.

Dimension	Specific indicator	Privileging criteria	Weighting (%)
Physician qualifications	Medical license and title	Valid medical license; Title ≥ Attending Physician	10%
	Clinical tenure	Continuous service in the department ≥ 2 years	5%
	Annual performance review	Average score ≥ 80 points in the last 2 years	5%
Surgical practice	Total surgical volume	≥ 80 procedures/2 years	15%
	Grade II–IV procedure volume	≥ 40 procedures/2 years	10%
	Surgical success rate	≥ 95%	10%
	Complication rate	< 5%	10%
Patient safety	Mean intraoperative blood loss	≤ Mean + 1SD for comparable procedures	8%
	Mean operative duration	≤ Mean + 15% for comparable procedures	7%
	Unplanned reoperation rate	< 1%	5%
	30-day readmission rate	< 5% (National baseline ≈ 2.13%)	5%
Academic contribution and collaboration	Research and teaching activity	Published ≥ 1 paper or undertaken ≥ 1 teaching task in past 2 years	3%
	Interdisciplinary collaboration	Multidisciplinary assessment score ≥ 80 points	2%

Surgery grades are categorized as: Grade I (simple), Grade II (moderately complex), Grade III (high complexity), and Grade IV (major procedures with significant risk). The ‘Medical License and Title’ indicator consolidates legal and seniority requirements into a single foundational qualification metric to streamline the assessment of baseline eligibility.

All indicators were automatically extracted and updated in real time through the health information system. A composite score (S) was computed using the weighted linear combination formula: $S=\sum (w_{i}\times s_{i})$ , where $\;w_{i}$ is the weight assigned via AHP and $s_{i}$ is the normalized score (0–100) for each indicator. The minimum threshold of 80 was established based on a retrospective analysis of the hospital’s historical top-quartile performers. The weighting system (Table 1) ensures that clinical safety and practice domains (totaling 70% of the total weight) act as the primary filters; due to this distribution, a surgeon with substandard safety metrics (e.g., high complication or reoperation rates) is mathematically precluded from reaching the 80-point threshold, regardless of their academic or seniority scores. This design directly drives evidence-based decision-making by prioritizing clinical competency over administrative status.

#### 2.2.2 Core features of the informatics platform

##### 2.2.2.1 Data integration module

Interfaced with HIS (Hospital Information System), EMR (Electronic Medical Record), PACS (Picture Archiving and Communication System), and anesthesia records to automatically capture key variables, providing the raw data foundation for indicators across all four dimensions (Physician Qualifications, Surgical Practice, Patient Safety, and Academic Contribution and Collaboration). This ensures that metrics such as surgeon demographics, case volume, blood loss, and research outputs are gathered with end-to-end authenticity and traceability.

##### 2.2.2.2 Multidimensional evaluation module

Embedded with the refined criteria, this module serves as the primary engine for scoring and decision-making. It applies the weighted composite algorithm to indicators within the four dimensions—calculating scores for credentials, practice volume, safety outcomes, and collaboration levels—to generate a standardized individual privileging index. It supports tiered privilege assignment (e.g., Levels I–III) based on these calculated index thresholds.

##### 2.2.2.3 Dynamic monitoring and alerting module

Focused on the Surgical Practice and Patient Safety dimensions, this module continuously tracks high-risk metrics—such as complication rates, unplanned reoperations, and abnormal blood loss—against the predefined performance cut-offs. Automatic alerts are triggered to the department chair and medical administration upon violation. As the central intervention of this study, the platform’s performance reporting was integrated into the administrative workflow. Monthly reports were submitted to the Medical Affairs Committee, and department chairs conducted mandatory reviews and coaching for surgeons with scores below 80 or safety alerts. This reporting-and-intervention loop allowed for measurable performance reassessment to confirm continuous improvement.

##### 2.2.2.4 Visualization and audit trail module

**Figure 1:**
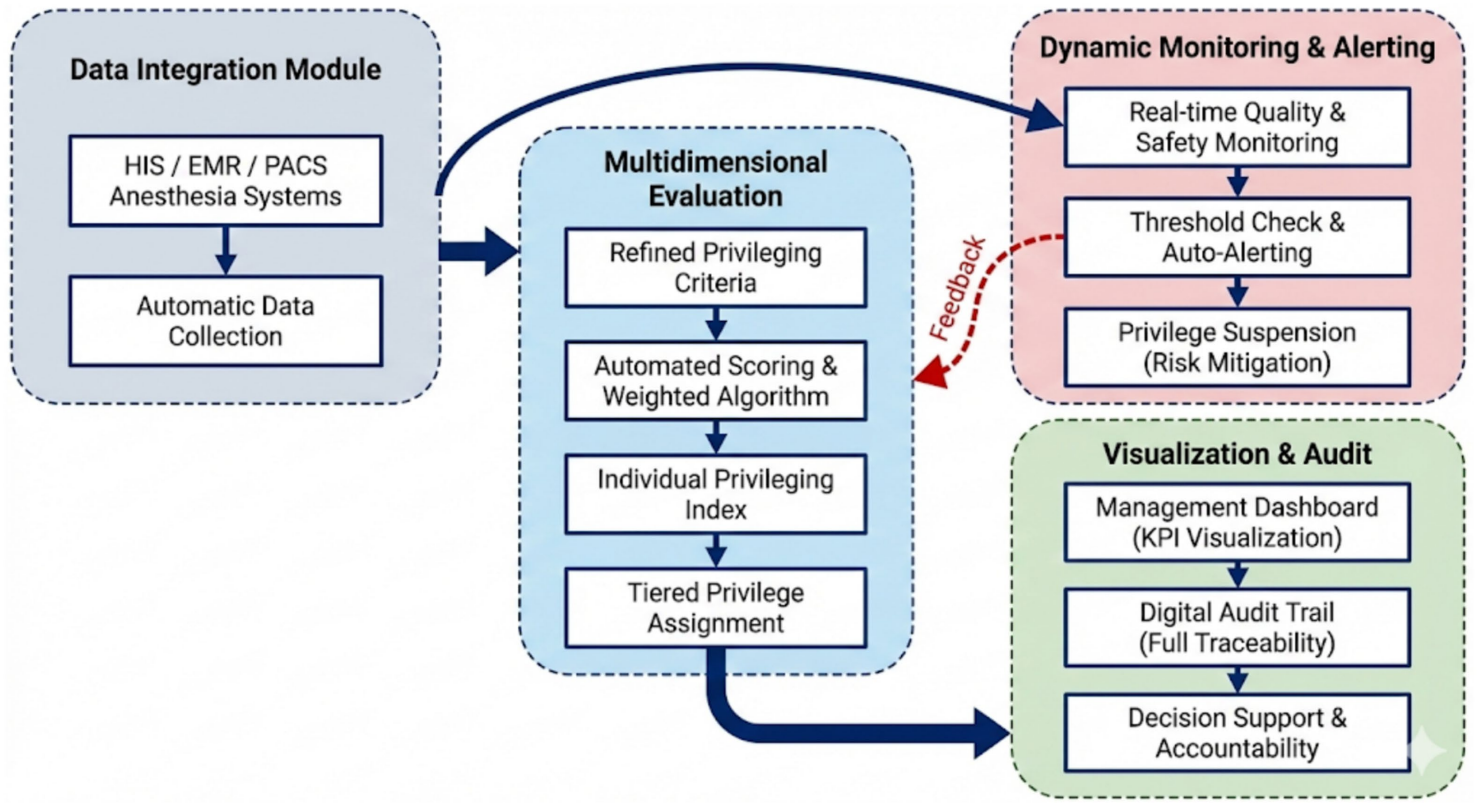
Functional module.

This module aggregates and visualizes the evaluation results from all four dimensions via a management dashboard. By presenting surgeon privileging indices, multi-dimensional performance trends, and risk profiles, it provides administrators with a comprehensive evidence base to support final authorization decisions and accountability. A complete digital audit trail—including scores, review comments, and privilege modifications—is maintained to ensure transparency and accountability. The functional architecture of the informatics platform, illustrating the closed-loop flow from data integration to dynamic monitoring, is shown in Figure 1.

#### 2.2.3 Management system architecture

**Figure 2:**
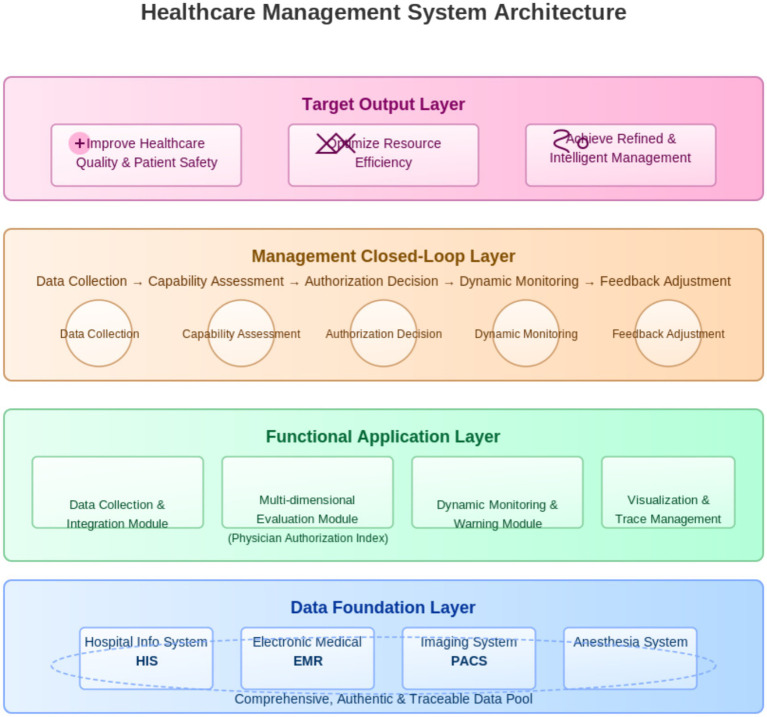
Management model architecture.

The model is structured into four hierarchical layers to ensure a data-driven, closed-loop workflow (Figure 2).

(1) Data Foundation Layer: Integrates the Hospital Information System (HIS), Electronic Medical Record (EMR), Picture Archiving and Communication System (PACS), and anesthesia systems to form a comprehensive, authentic, and traceable data pool.

(2) Functional Application Layer: Comprises the four core modules described in Section 1.2.2, serving as the technical engine for evaluation and monitoring.

(3) Management Closed-Loop Layer: Facilitates the end-to-end administrative workflow, progressing from data acquisition and capability assessment to authorization decisions, dynamic monitoring, and feedback adjustment.

(4) Target Output Layer: Aims to achieve the overarching objectives of enhanced medical quality, optimized resource allocation, and intelligent surgical management.

### 2.3 Evaluation framework for system effectiveness

This section defines the outcomes used to measure the model’s overall impact. While the four-dimensional criteria in Table 1 function as the “input” (measuring individual surgeon competency to determine privileges), the following five dimensions serve as the “output” (evaluating the institutional effectiveness of the new model): Process Efficiency, Clinical Quality and Safety, Resource Utilization, System Performance, and Satisfaction.

#### 2.3.1 Process efficiency indicators

Process efficiency metrics were employed to assess the timeliness and operational fluency of the surgical privileging process. Three core indicators were established: privileging review turnaround time, information processing completion rate, and exception resolution rate. The informatics platform automatically captured the end-to-end duration from application submission to final approval, while concurrently monitoring completion statuses for document submission, data validation, and privilege activation. Applications requiring resubmission or modification were tracked to quantify exceptions and their subsequent resolution. The system generated automated weekly summaries, monthly analytical reports, and quarterly consolidated analyses. Performance benchmarks were defined as follows: review duration ≤5 working days (satisfactory), >7 days (unsatisfactory); information processing rate ≥95% (efficient), 90–95% (moderate), <90% (inefficient); exception resolution rate ≥85% (compliant), <70% (indicating substantial process deficiencies).

#### 2.3.2 Clinical quality and safety indicators

These indicators evaluated the impact of the privileging framework on clinical outcomes and patient safety. Four core metrics were implemented: procedural success rate, postoperative complication incidence, unplanned reoperation rate, and mortality. Data were automatically extracted from operative reports, electronic health records, and follow-up systems, with validation by medical record quality committees. Procedural success was defined as the attainment of intended surgical objectives; complications were classified per the Clavien-Dindo system; unplanned reoperations and 30-day readmissions were tracked via the hospital information system. Metrics were aggregated monthly, analyzed quarterly, and compared annually. Performance standards were derived from National Surgical Quality Improvement Program (NSQIP) benchmarks and regional healthcare administrative standards (1, 4). Specifically: procedural success rate ≥95% (excellent); complication rate ≤10% (aligned with specialty-specific norms); 30-day readmission rate <5% (calibrated against the national baseline of ~2.13% to account for complex referral cases); and mortality rate ≤1% (defined as the maximum tolerable threshold for general surgical safety review).

#### 2.3.3 Resource utilization indicators

Resource utilization metrics assessed the model’s impact on operational efficiency and resource allocation. Three key indicators were adopted: operating theater turnover rate, average length of stay (ALOS), and per-procedure supply costs. Turnover rate was derived from inter-procedure intervals recorded in anesthesia documentation; ALOS was automatically calculated from admission/discharge timestamps; supply costs were obtained from hospital cost-accounting systems. Data were compiled weekly, aggregated monthly, and subjected to quarterly trend analysis. Benchmark values were established as: turnover rate ≥85% (efficient), 70–85% (moderate), <70% (inefficient); ALOS ≤9 days (compliant), >12 days (unsatisfactory); supply costs within ±10% of specialty-specific averages (acceptable), >20% above average (triggering utilization review).

#### 2.3.4 System performance indicators

System performance indicators were utilized to evaluate the operational effectiveness and user satisfaction of the information-enabled platform in surgical authorization management, ensuring technical reliability and scalability. Three core metrics were established: system operational stability, data processing timeliness, and user satisfaction. System stability was automatically monitored through system logs recording the number of downtime incidents and mean time to repair (MTTR). Data processing timeliness was assessed by comparing simulated tests with actual operational data, measuring the average duration from data collection to result generation. User satisfaction was evaluated using a 5-point Likert scale questionnaire covering interface usability, operational convenience, and decision-support effectiveness. System operation and data processing were monitored monthly, while user satisfaction was surveyed semi-annually. Performance thresholds were defined as follows: system stability required ≤1 downtime incident per quarter and MTTR <2 h; data processing time ≤10 min was considered compliant, while >20 min was non-compliant; user satisfaction scores ≥4.0 were classified as excellent, 3.0–4.0 as acceptable, and <3.0 indicated need for improvement.

#### 2.3.5 Satisfaction indicators

Satisfaction indicators were employed to assess the acceptability and implementation potential of the surgical authorization management model in clinical practice, reflecting the subjective experiences of physicians, patients, and administrators. Three core metrics were defined: physician satisfaction, patient satisfaction, and administrator satisfaction. Evaluation was conducted through a combination of questionnaires and structured interviews. Physician satisfaction encompassed transparency of the authorization process, operational convenience, and professional development support; patient satisfaction included perceived surgical safety, waiting time, and overall care experience; administrator satisfaction covered decision-making efficiency, regulatory controllability, and cost-effectiveness. All questionnaires used a 5-point Likert scale, with 1 indicating “very dissatisfied” and 5 “very satisfied.” Physician and patient satisfaction were surveyed quarterly, while administrator satisfaction was assessed semi-annually. Mean scores ≥4.0 were considered excellent, 3.5–4.0 acceptable, and <3.5 indicated need for optimization.

### 2.4 Data analysis

Data analysis was performed using SPSS 26.0. Continuous variables were expressed as mean ± standard deviation (x̄ ± SD), while categorical variables were presented as frequencies and percentages. Within-group comparisons were conducted using *t*-tests and chi-square tests (χ^2^). For categorical variables with rare events (expected frequency < 5), Fisher’s exact test was employed. To address the potential for “overpowered” significance due to the large patient sample size (*N* = 3,914), effect sizes were calculated: Cohen’s d for continuous variables and Cramer’s V for categorical data, with values interpreted as small, medium, or large based on standard benchmarks.

Between-group comparisons were performed using independent samples *t*-tests or analysis of variance (ANOVA); non-normally distributed data were expressed as median (interquartile range) [M (P25, P75)] and analyzed using Mann–Whitney U tests or Kruskal-Wallis H tests. For multidimensional evaluation indicators, a comprehensive scoring system was constructed and analyzed using ANOVA or Kruskal-Wallis tests to examine differences among physician groups across process efficiency, medical quality, resource utilization, system performance, and satisfaction metrics. All statistical tests were two-sided, with *p* < 0.05 considered statistically significant.

## 3 Results

### 3.1 Baseline characteristics of study subjects

Baseline data indicated that the majority of medical staff were male (73.4%), with 82.3% holding the title of attending physician or higher. The mean age was 38.72 ± 6.21 years, with an average seniority of 11.34 ± 4.57 years and a historical total surgical volume of 212.45 ± 48.33 procedures. The mean patient age was 54.28 ± 12.47 years. Comparison across surgical specialties indicated that gender, disease complexity, and ASA score distributions were balanced (*p* > 0.05). While the distribution of professional titles showed a marginal statistical difference (*p* = 0.041), all metrics—including titles—exhibited small effect sizes (Cramér’s V < 0.225), confirming the overall baseline comparability of the sample across the study period (Table 2).

**Table 2:** Baseline characteristics of study subjects.

Category	Indicator	Subjects/patients (n)	Mean ± SD/n (%)	*t*/χ^2^	*P*	Effect size
Medical staff	Age (years)	79	38.72 ± 6.21	–	–	–
Medical staff	Male, n (%)	79	58 (73.4%)	0.002	0.964	0.029 (Cramér’s V)
Medical staff	Title ≥ attending physician, n (%)	79	65 (82.3%)	4.178	0.041	0.225 (Cramér’s V)
Medical staff	Years of experience	79	11.34 ± 4.57	–	–	–
Medical Staff	Total historical surgical volume	79	212.45 ± 48.33	–	–	–
Patients	Age (years)	3,914	54.28 ± 12.47	–	–	–
Patients	Male, n (%)	3,914	2,205 (56.4%)	0.467	0.494	0.001 (Cramér’s V)
Patients	Disease Complexity ≥ Moderate, n (%)	3,914	1,387 (35.4%)	1.832	0.176	0.070 (Cramér’s V)
Patients	ASA Score≥III	3,914	542 (13.8%)	2.107	0.147	0.023 (Cramér’s V)

ASA, American Society of Anesthesiologists physical status classification.

Application of the criteria from Table 1 revealed that the mean baseline composite score for surgeons was 86.42 ± 5.17. Notably, 6 surgeons (7.6%) initially failed to reach the 80-point threshold for independent Grade III–IV procedure privileges, primarily due to low scores in the “Patient Safety” and “Surgical Practice” dimensions. Following targeted interventions facilitated by the informatics platform, 4 of these individuals (66.7%) achieved passing scores in subsequent evaluations.

### 3.2 Process efficiency

Compared with the pre-implementation phase, the review time significantly decreased from 8.64 ± 2.15 days to 4.85 ± 1.15 days in Q1 (*p* < 0.001), while the information transfer completion rate and exception process correction rate significantly improved from 78.42 and 65.24% to over 95 and 86%, respectively (*p* < 0.001). Following implementation, these indicators remained stable across the four quarters, ranging from 4.61 to 4.85 days, 95.87–96.91%, and 86.92–88.12%, respectively. No statistically significant differences were observed between the four post-implementation quarters (*p* > 0.05) (Table 3).

**Table 3:** Process efficiency indicators comparing pre- and post-implementation.

Indicator	Pre-implementation (mean ± SD)	Q1 (post-implementation)	Q2 (post-implementation)	Q3 (post-implementation)	Q4 (post-implementation)	*F*	*P*
Review time (workdays)	8.64 ± 2.15	4.85 ± 1.15	4.73 ± 1.12	4.68 ± 1.09	4.61 ± 1.08	1.273	0.288
Information transfer completion rate (%)	78.42 ± 7.31	95.87 ± 3.22	96.42 ± 3.17	96.58 ± 3.05	96.91 ± 3.01	1.642	0.184
Exception process correction rate (%)	65.24 ± 8.42	86.92 ± 5.35	87.56 ± 5.24	87.88 ± 5.18	88.12 ± 5.11	1.517	0.205

### 3.3 Medical quality and safety

Compared with the pre-implementation baseline, the surgical success rate significantly improved from 95.14% to over 98% (*P*_a_ < 0.01), while key safety indicators—including postoperative complications, 30-day readmissions, and unplanned reoperations—showed marked reductions (*P*_a_ < 0.05). Following the initial implementation, the surgical success rate remained consistently high (ranging from 97.82 to 98.68%), and other safety metrics exhibited long-term stability. Fisher’s exact tests (for rare events) and chi-square tests confirmed no statistically significant differences across the four post-implementation quarters (*P*_b_ > 0.05), demonstrating that the improvements in medical quality and patient safety were effectively sustained (Table 4).

**Table 4:** Medical quality and safety indicators (pre- vs. post-implementation).

Indicator	Pre-impl (%)	Q1 (%)	Q2 (%)	Q3 (%)	Q4 (%)	P_a_ (pre vs. post)	P_b_ (among Q1–Q4)
Surgical success rate	**95.14**	97.82	98.46	98.68	98.12	**<0.01**	**0.078**
Postoperative complications	**1.42**	0.21	0.2	0.2	0.16	**<0.001**	**0.292**
30-day readmission rate	**2.34**	1.89	1.63	1.54	1.21	**<0.05**	**0.433**
Unplanned reoperation	**2.15**	1.03	1.01	0.98	0.95	**<0.01**	**0.315**

Bold values denote *p*-values for comparisons among Q1–Q4.

### 3.4 Resource utilization

The operating room turnover rate, average length of stay, and per capita surgical supply cost remained stable throughout the four quarters, ranging from 84.12 to 86.58%, 8.91 to 9.12 days, and 3085.24 to 3125.48 RMB, respectively. No statistically significant differences were observed between quarters for any of these indicators (*p* > 0.05) (Table 5).

**Table 5:** Resource utilization indicators.

Indicator	Q1 (mean ± SD)	Q2 (mean ± SD)	Q3 (mean ± SD)	Q4 (mean ± SD)	*F*	*P*
Operating room turnover rate (%)	84.12 ± 5.21	85.46 ± 5.13	86.02 ± 5.08	86.58 ± 5.01	2.134	0.089
Average length of Stay (days)	9.12 ± 1.47	9.05 ± 1.42	8.98 ± 1.38	8.91 ± 1.36	1.327	0.269
Per capita surgical supply cost (CNY)	3125.48 ± 425.36	3112.75 ± 418.29	3098.62 ± 412.11	3085.24 ± 407.58	1.142	0.336

CNY = Chinese Yuan (RMB).

### 3.5 System performance metrics

System operational stability, data processing timeliness, and user satisfaction were maintained at excellent levels throughout the study period. The number of system downtime incidents was ≤1 per quarter, mean time to repair was <2 h, processing time was ≤10 min, and user satisfaction scores ranged from 4.12 to 4.26 points. No statistically significant differences were observed between quarters for any performance metrics (*p* > 0.05) (Table 6).

**Table 6:** System performance.

Indicator	Q1 (mean ± SD)	Q2 (mean ± SD)	Q3 (mean ± SD)	Q4 (mean ± SD)	*F*	*P*
System downtime (times/quarter)	0.92 ± 0.28	0.85 ± 0.25	0.88 ± 0.26	0.81 ± 0.23	1.643	0.183
Mean time to repair (hours)	1.73 ± 0.42	1.68 ± 0.38	1.65 ± 0.36	1.62 ± 0.34	1.291	0.271
Data processing timeliness (minutes)	9.48 ± 1.32	9.32 ± 1.28	9.21 ± 1.25	9.08 ± 1.22	1.374	0.256
User satisfaction (points)	4.12 ± 0.41	4.18 ± 0.39	4.21 ± 0.38	4.26 ± 0.36	2.071	0.12

### 3.6 Satisfaction

Satisfaction levels among physicians, patients, and administrators remained consistently high across all quarters, with no statistically significant differences observed (p > 0.05). These results indicate strong acceptance of the surgical privilege management model and support its sustainable implementation (Table 7).

**Table 7:** Satisfaction.

Indicator	Q1 (mean ± SD)	Q2 (mean ± SD)	Q3 (mean ± SD)	Q4 (mean ± SD)	*F*	*P*
Physician satisfaction (points)	4.12 ± 0.32	4.18 ± 0.31	4.21 ± 0.29	4.25 ± 0.28	2.014	0.129
Patient satisfaction (points)	4.08 ± 0.36	4.13 ± 0.34	4.16 ± 0.33	4.21 ± 0.32	1.872	0.152
Administrator satisfaction (points)	–	4.11 ± 0.39	–	4.19 ± 0.35	1.954	0.14

### 3.7 System stability and performance

**Figure 3:**
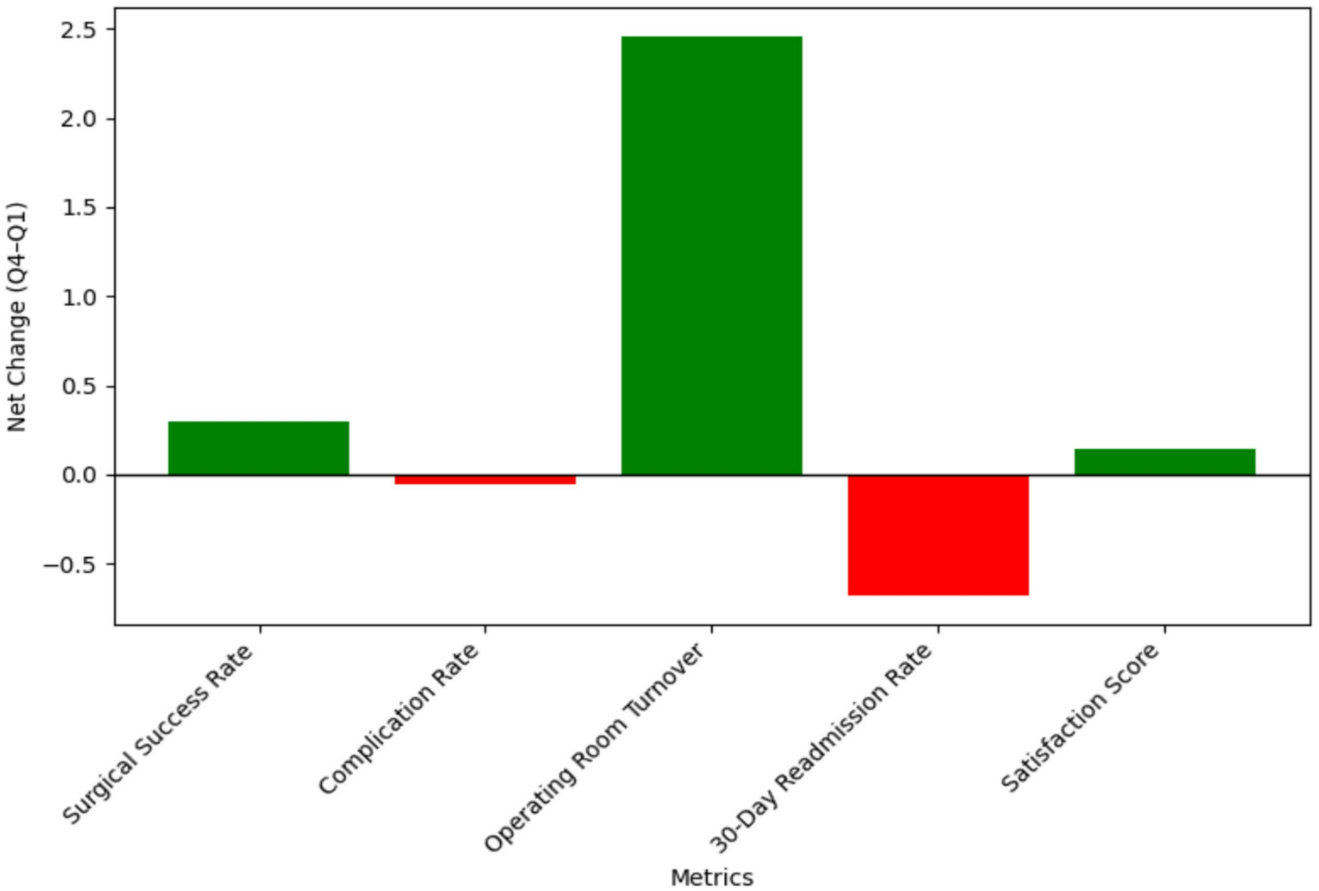
System performance and clinical quality (contribution of quarterly change). Displays the contribution of quarterly change by metric. The bars represent the net change from Q1 to Q4 for each indicator, with green bars showing improvements and red bars indicating slight regressions.

**Figure 4:**
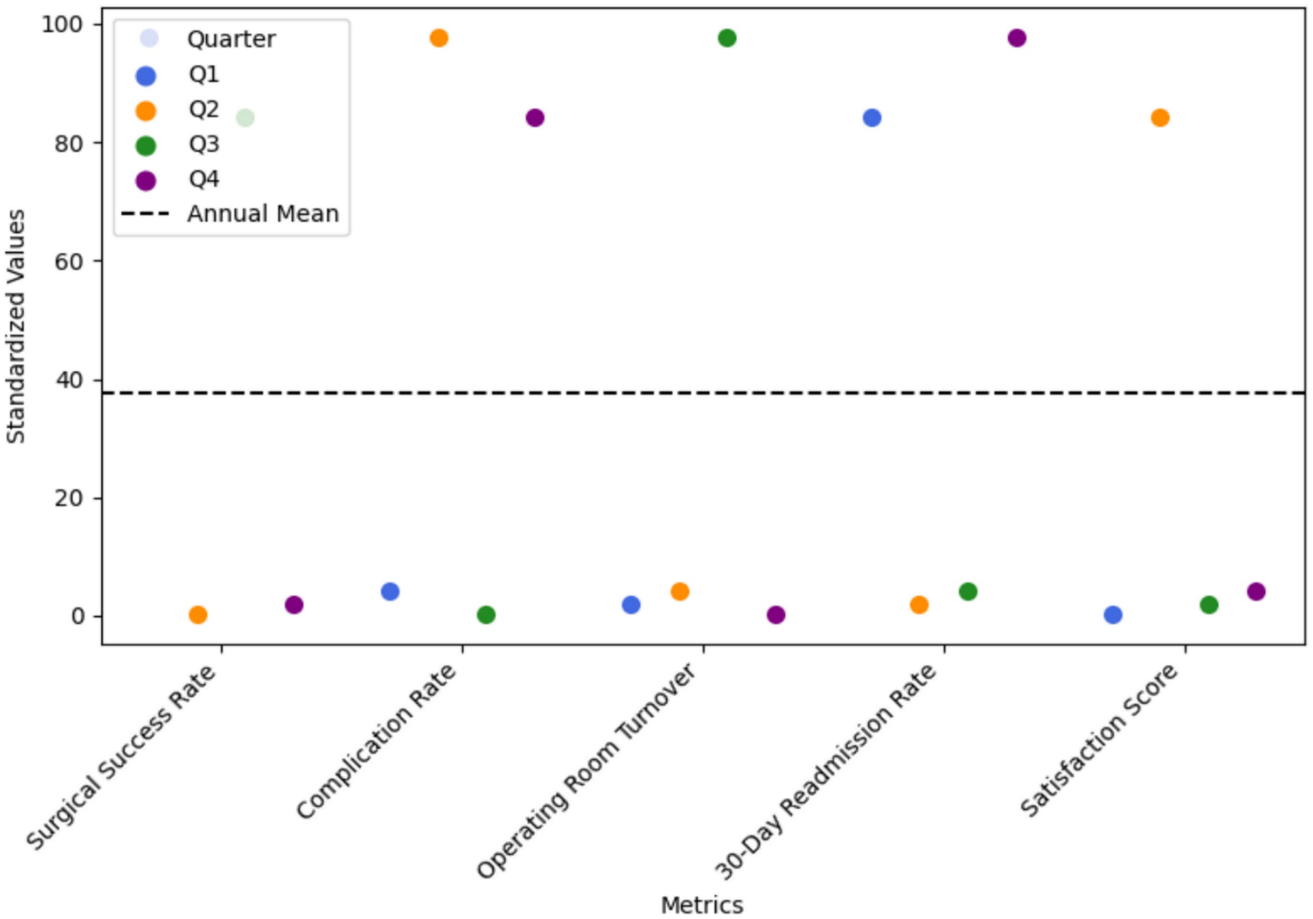
System performance and clinical quality (quarterly standardized values). A beeswarm plot illustrating the quarterly values (standardized) for each metric, with different colors for each quarter (Q1: Royal blue, Q2: Deep orange, Q3: Forest green, Q4: Purple).

Overall, most indicators related to efficiency, safety, resource utilization, and satisfaction remained stable throughout the four quarters. A limited number of metrics, including surgical supply costs and review duration, exhibited modest reductions. Minor positive contributions were observed, reflecting incremental improvements in satisfaction and slight enhancements in process and safety stability. As illustrated in Figure 3, the net change (Q4–Q1) for most indicators hovered near zero, which—given the significant initial improvements—confirmed that the system’s performance and clinical quality were consistently maintained at the high levels achieved post-implementation, without significant fluctuations. The standardized quarterly trends of all metrics, presented in Figure 4, further demonstrate the stability across all observation periods.

Detailed representative case analyses illustrating the platform’s performance across complex elective, emergent, and high-risk geriatric procedures are provided in Supplementary material.

## 4 Discussion

This study constructed and empirically evaluated an informatics-driven refined surgical privileging model, demonstrating its integrated value in improving operational quality, safeguarding patient safety, and optimizing resource utilization. Results indicated that all core metrics remained consistently high throughout the four-quarter observation period. Importantly, although the large sample size (*N* = 3,914) theoretically increases the risk of “overpowered” statistical significance, our use of effect size analysis (Cramér’s *V* < 0.1) confirms that the inter-quarter variations were negligible, further validating the stability and reliability of the platform’s performance over time (8).

These outcomes present a notable contrast to traditional privileging systems reliant on administrative experience or seniority-based criteria (10). Previous research has predominantly focused on unidimensional or static authorization standards (11, 12). Our findings contrast with these models by demonstrating that a multidimensional approach can sustain an extremely low complication rate (0.16–0.21%) and a high success rate (98.68%), which are more favorable than the outcomes seen in traditional systems where safety monitoring is often decoupled from the privileging process (10, 13). Innovatively, this study introduced a multidimensional evaluation system covering four domains: surgical qualifications, operational experience, patient safety, and interdisciplinary contribution. By implementing an informatics platform enabling real-time data capture, dynamic weighting, and automated scoring, the model effectively mitigates inherent shortcomings of conventional methods (14). Specifically, the 44.3% reduction in review turnaround time (from 8.64 to 4.81 days) observed in this study represents a more substantial efficiency gain compared to the 20–30% optimization reported in earlier clinical pathway integration studies (4), suggesting that a dedicated privileging engine provides superior administrative agility.

From an evidence-construction perspective, this study reinforces that information empowerment serves as the foundational mechanism for refining surgical privileging. The data integration module interfaces natively with hospital information systems—including HIS, EEMR, PACS, and anesthesia systems—to achieve holistic and interoperable surgical data collection, ensuring accuracy and traceability (15, 16). The multidimensional evaluation module utilizes weighted algorithms and adaptive thresholding to transform abstract surgical capability into quantifiable privileging indices, offering an objective basis for managerial decision-making (17). Through continuous monitoring of real-time risk indicators such as complication rates and operative time, the dynamic alerting module facilitates immediate privilege adjustments and preemptive risk mitigation, effectively addressing regulatory gaps post-authorization (13). Furthermore, the visualization and audit-trail module enhances procedural transparency and strengthens surgeon trust in the privileging process. The synergistic operation of these modules constitutes an end-to-end closed-loop management system encompassing data acquisition, evaluation, decision-making, monitoring, and refinement. Its efficacy is further validated through case-based analyses (18), which illustrate the model’s adaptability and controllability across diverse scenarios—complex elective surgeries, emergent operations, and high-risk geriatric procedures. Significant improvements were observed in authorization turnaround time, reduction of complications, and stakeholder satisfaction. These positive trends reinforce the value of “risk-tailored” management as advocated by Klatte et al. (8), but go further by proving that a dynamic weighting system can successfully translate high-level management objectives into stable, quantifiable clinical performance across diverse surgical specialties.

Compared with earlier theoretical work, this model exemplifies the concrete application of information-enablement theory in healthcare management. While conventional management frameworks emphasize standardization and process refinement, most remain confined to policy design and fail to adequately resolve information asymmetry and implementation variability (19, 20). This study translates management protocols into executable digital rules through algorithmic embedding, thereby diminishing human intervention and subjective bias (21). This mechanism aligns not only with core tenets of lean management—waste reduction and efficiency enhancement—but also resonates with complex adaptive systems theory wherein informational feedback enables self-organization and continuous optimization (8). Furthermore, the established reporting-and-intervention mechanism promotes physician behavior change by creating structured feedback loops consistent with “Control Theory.” By integrating quantified performance reports into administrative workflows followed by clinical coaching, the model establishes a direct cognitive link between clinical practice and privileging outcomes. The visualization of specific performance gaps—such as safety alerts or scores below the threshold —serves as a stimulus for self-regulation and intentional behavior modification. The significant improvement in scores among previously substandard surgeons, with 66.7% reaching passing thresholds after intervention, demonstrates that this informatics-driven feedback loop effectively transforms passive monitoring into active professional development and sustained clinical safety. Most significantly, by adopting a data-driven rather than experience-driven approach, this model enables a paradigm shift in surgical management from static credentialing toward dynamic competency-based privileging.

While this study provides robust evidence supporting the informatics-driven model, several limitations should be acknowledged. First, its single-center design and purposive sampling of surgeons meeting high volume thresholds may limit the generalizability of the findings and potentially overestimate the effect size in broader, less-selected populations. Second, the observed improvements could be partially attributable to the Hawthorne effect, as participants were aware of the new monitoring system. Third, the model was implemented in a tertiary hospital with advanced digital infrastructure; its feasibility and effectiveness in resource-limited settings require further validation. Additionally, we did not conduct a formal cost–benefit analysis of the platform’s development and maintenance, which is crucial for hospital administrators considering adoption. Finally, unmeasured confounding factors, such as concurrent hospital-wide quality initiatives, may have contributed to the outcomes.

In conclusion, this empirical investigation validates the comprehensive value of an informatics-refined surgical privileging model in elevating clinical quality, enhancing patient safety, and improving resource allocation. It addresses critical deficiencies in conventional approaches—including subjectivity, latency, and insufficient transparency—while achieving precision, intelligence, and closed-loop control through integrated data processing, dynamic evaluation, and real-time monitoring. The model’s successful implementation provides a transferable theoretical framework and methodological foundation for contemporary surgical management, promoting a fundamental transition from experience-based to scientific, and from coarse to refined, operational paradigms. It offers a practical and scalable pathway toward institutional high-quality development and reinforced patient safety.

Future investigations should therefore expand to multi-center, pragmatic trials with more diverse surgeon populations to enhance external validity. Longitudinal studies extending beyond 1 year are needed to assess the sustainability of outcomes and long-term impact on surgeon development. Furthermore, research should quantify the total cost of ownership and return on investment of such platforms. Finally, exploring the deeper integration of artificial intelligence for predictive risk analytics and fully automated, dynamic privilege adjustment represents a promising frontier for next-generation surgical management systems.

## Data Availability

The original contributions presented in the study are included in the article/Supplementary material, further inquiries can be directed to the corresponding authors.
