# Supplementary material for: Digital empowerment for refined surgical privileging management: a model construction and empirical study
Source: Front Public Health. 2026 Mar 24;14:1701167. doi: 10.3389/fpubh.2026.1701167 (PMC13055508; doi:10.3389/fpubh.2026.1701167)
Supplement: Supplementary file 1 [file Table_1.DOCX]

**Representative Case Analyses**
**Case 1:** A 65-year-old male patient undergoing complex hepatobiliary surgery, with comorbid hypertension and ASA grade III. The privilege request was processed through the informatics platform in 3 days, with a 100% information transfer completion rate and zero exception corrections required. The procedure achieved 100% success with no postoperative complications. The length of stay was 9 days, and supply costs remained within ±5% of the average for comparable procedures. Satisfaction scores were 5.00 across physicians, patients, and administrators, demonstrating the platform’s efficient management of complex cases.

**Case 2:** A 58-year-old female patient requiring emergency cardiac surgery, classified as ASA grade III. Privilege authorization was completed within 2 days, with a 95% information transfer completion rate and one exception correction. The surgery was successful with no complications. The average length of stay was 8 days, and supply costs were 8% above the average for similar procedures. Satisfaction ratings were 4.53 (physicians), 4.80 (patients), and 4.60 (administrators), indicating the platform’s responsiveness in emergency settings.

**Case 3:** A 78-year-old female patient undergoing abdominal tumor surgery, with multiple chronic conditions and ASA grade IV. The privilege review process required 4 days, with a 92% information transfer completion rate and two exception corrections. The procedure was successful, although a mild complication (Clavien-Dindo Grade II) occurred. The length of stay was 12 days, with supply costs within acceptable limits. Satisfaction scores were 4.20 (physicians), 4.02 (patients), and 4.13 (administrators), reflecting the platform’s controllability and safety in high-risk surgical management.
